# Reduction of T-Box 15 gene expression in tumor tissue is a prognostic biomarker for patients with hepatocellular carcinoma

**DOI:** 10.18632/oncotarget.27852

**Published:** 2020-12-29

**Authors:** Yuji Morine, Tohru Utsunomiya, Yu Saito, Shinichiro Yamada, Satoru Imura, Tetsuya Ikemoto, Akihiro Kitagawa, Yuta Kobayashi, Seiichiro Takao, Keisuke Kosai, Koshi Mimori, Yasuhito Tanaka, Mitsuo Shimada

**Affiliations:** ^1^Department of Surgery, Institute of Biomedical Sciences, Tokushima University Graduate School, Tokushima 770-8503, Japan; ^2^Department of Surgery, Kyushu University Beppu Hospital, Beppu 874-0838, Japan; ^3^Department of Gastroenterology and Hepatology, Faculty of Life Sciences, Kumamoto University, Kumamoto 860-8556, Japan

**Keywords:** genome-wide analysis, methylation, tumor suppressor gene, prognostic biomarker, hepatocellular carcinoma

## Abstract

Genome-wide analysis is widely applied to detect molecular alterations during oncogenesis and tumor progression. We analyzed DNA methylation profiles of hepatocellular carcinoma (HCC), and investigated the clinical role of most heypermethylated of tumor, encodes T-box 15 (TBX15), which was originally involved in mesodermal differentiation. We conducted a genome-wide analysis of DNA methylation of tumor and non-tumor tissue of 15 patients with HCC, and revealed *TBX15* was the most hypermethylated gene of tumor (Beta-value in tumor tissue = 0.52 compared with non-tumor tissue). Another validation set, which comprised 58 HCC with radical resection, was analyzed to investigate the relationships between tumor phenotype and *TBX15* mRNA expression. *TBX15* mRNA levels in tumor tissues were significantly lower compared with those of nontumor tissues (*p* < 0.0001). When we assigned a cutoff value = 0.5-fold, the overall survival 5-year survival rates of the low-expression group (*n* = 17) were significantly shorter compared with those of the high-expression group (*n* = 41) (43.3% vs. 86.2%, *p* = 0.001). Multivariate analysis identified low *TBX15* expression as an independent prognostic factor for overall and disease-free survival. Therefore, genome-wide DNA methylation profiling indicates that hypermethylation and reduced expression of *TBX15* in tumor tissue represents a potential biomarker for predicting poor survival of patients with HCC.

## INTRODUCTION

Hepatocellular carcinoma (HCC) is the sixth most prevalent human cancer and third-highest cause of cancer-related deaths worldwide, and its incidence is significantly increasing [1, 2]. Despite advances in surgical techniques, radiofrequency ablation, and trans-arterial therapy, the prognosis of HCC patients is unacceptable because of post-treatment relapse and distant metastasis. Therefore, great efforts have been made to identify novel prognostic factors for managing patients with HCC as well as molecular targets to prevent oncogenesis and tumor progression [3–6].

Several genome-wide studies of the molecular alterations in tumor and non-tumor liver tissues addressed DNA, mRNA, and microRNA (miRNA) expression as well as epigenetic alterations to predict recurrence and hepatocarcinogenesis after treating patients with curative intent according to distinct gene expression or alteration patterns [7–11]. Here we focused on the epigenetic alterations of the genomes of tumor and non-tumor cells of patients with HCC to identify the mechanisms of tumor progression and hepatocarcinogenesis., because global DNA hypomethylation or cancer specific DNA hypermethylation occurs in certain carcinomas [12]. And we firstly chose long interspersed nuclear element-1 (LINE-1) sequences provided a surrogate marker of global DNA methylation levels, and detected hypomethylation of LINE-1 in HCC tissues, which was significantly associated with poor prognosis of patients with HCC through the activation of MET [13]. Further, we investigated epigenetic characteristics with array-based analysis of DNA methylation using the Illumina Human Methylation 450 BeadChip, and identified specific DNA methylation profiling in nontumor liver tissue of patients without HCV and HBV detectable infection (NBNC-HCC), which possibly contributed to the development of HCC [14].

In this study, we analyzed DNA methylation profiles in tumor tissue of HCC using those case series of our recent study [14], and found the most hypermethylated gene in tumor compared with nontumor tissues, encodes T-box 15 (TBX15), implicating TBX15 as a candidate regulator of tumor progression. Hence, the aim of the present study was to identify novel biomarkers for early diagnosis, risk assessment, and chemoprevention of HCC. For this purpose, we further evaluated the clinical significance of *TBX15* expression levels associated with tumor phenotype as well as with the prognosis of patients with HCC who underwent hepatectomy.

## RESULTS

### Detection of differentially methylated genes

**Figure 1:**
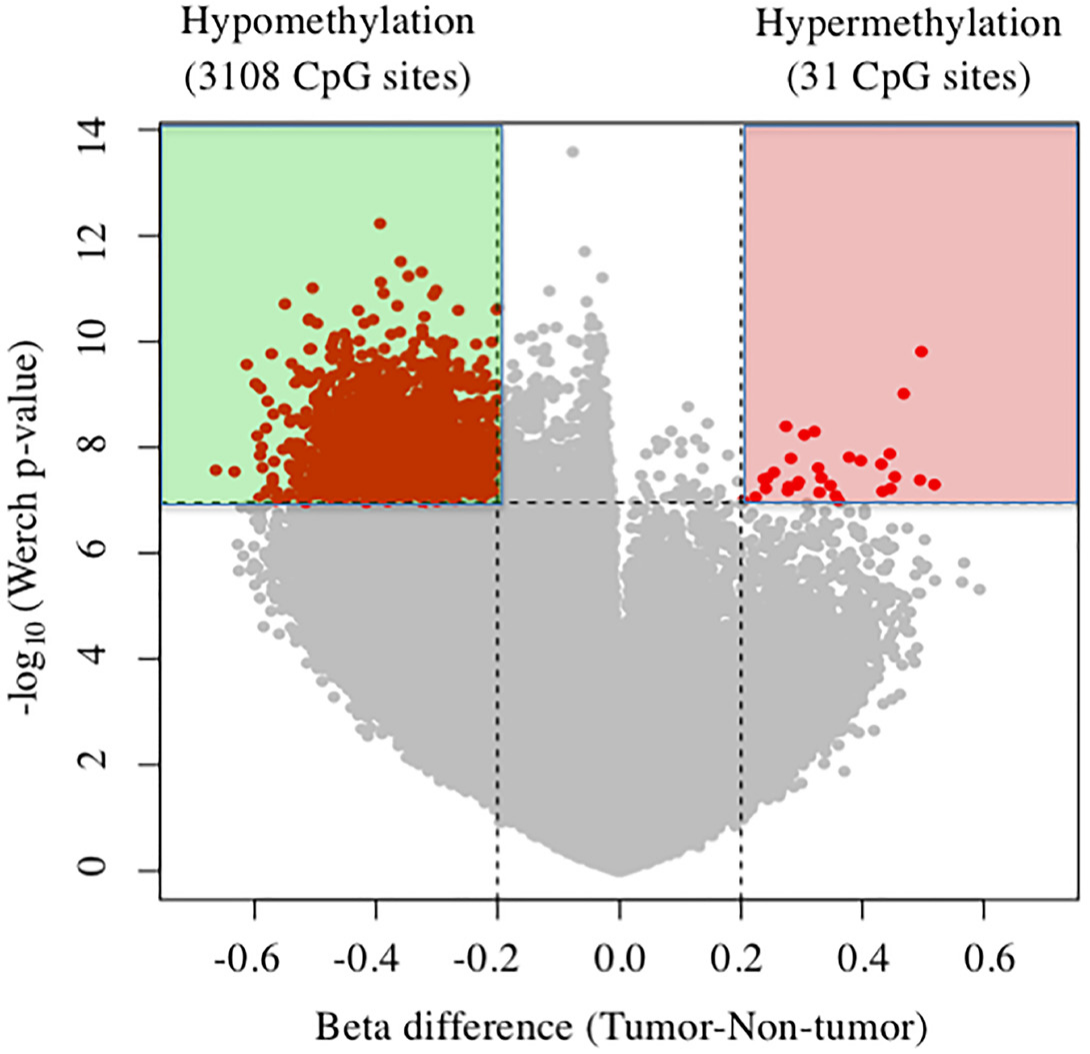
Volcano plots of DNA methylation in tumor tissues compared with nontumor tissue. Significant associations are indicated in red.

Figure 1 shows volcano plots of 3,139 differentially methylated CpG sites in tumor tissues compared with nontumor liver tissues (beta-value difference > 0.2, *P* < 0.05). Consistent with previous reports [15, 16], here we detected global differential DNA hypomethylation (3,108 CpG sites) in tumor tissues compared with nontumor tissues. Further, 31 CpG sites were hypermethylated, corresponding to 16 gene promoters that are commonly hypermethylated in tumor tissues compared with nontumor tissues (Table 1). Among them, *TBX15* of tumor tissue was the most hypermethylated (mean difference of beta-value = 0.518765).

**Table 1:** Genes with hypermethylated CpG sites in HCC tissues

Gene name	Target ID	Chromosome	Position	Mean difference
TBX15	cg10703826	1	119333639	0.518765
PITX1	cg00396667	5	134392286	0.446505
ILDR2	cg25147376	1	165156917	0.360129286
GRHL2	cg23973429	8	102573922	0.347427857
LOC146880	cg01720033	17	60208206	0.327155
RTN4R	cg12393104	22	18613758	0.320887143
INF2	cg07155724	14	104236775	0.304177857
CYB5R2	cg05919312	11	7650739	0.294705
GAB1	cg17453767	4	144497246	0.282047857
PITPNM2	cg01973483	12	122157076	0.278242143
C1QTNF4	cg18356785	11	47568356	0.277035714
KIAA0664	cg12229632	17	2554656	0.273881429
PPFIA3	cg08319905	19	54328082	0.254201429
HOXA10	cg10724867	7	27185392	0.242810714
PKP4	cg13168187	2	159231927	0.237381429
ANKS1A	cg07254055	6	35119746	0.203343571

To explore the relationship between *TBX15* methylation status and HCC tumor malignancy, we investigated the *TBX15* methylation analysis of HCC patients from TCGA data. Among those data, 59 registered CpG sites of *TBX15* were identified and could be classified into 3 clusters according to its beta-value (Supplementary Figure 1). Consequently, the high methylation beta-value of *TBX15* was significantly associated with poor disease-free survival in HCC patients, regardless of cluster classification, while poor overall survival only in cluster 3 (Supplementary Figure 2). Next, we investigated the *TBX15* mRNA expression in several cancers from TCGA. *TBX15* mRNA expression in tumor tissue was varied according to type of carcinoma, while it was significantly lower in tumor tissue of HCC compared to nontumor tissue (Supplementary Figure 3). Also, we found that the same results of HCC were obtained in breast cancer and cholangiocarcinoma.

**Figure 2:**
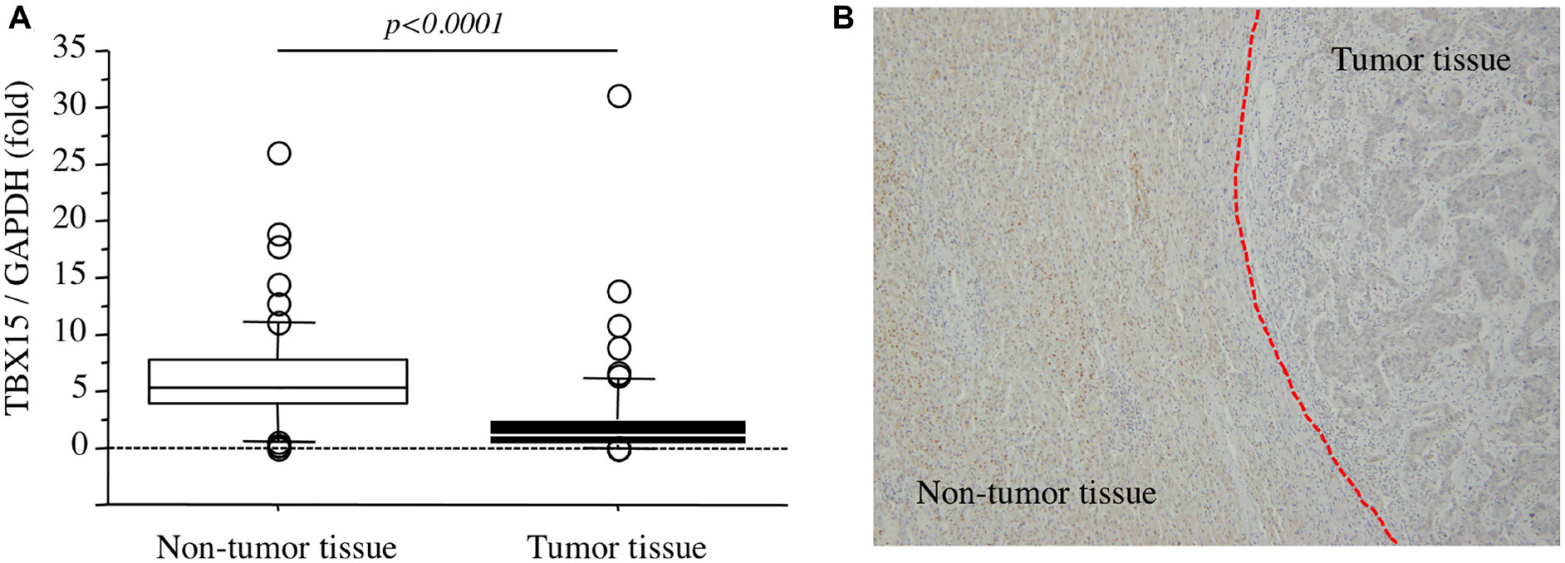
(**A**) *TBX15* mRNA expression in tumor tissues and nontumor tissues. (**B**) *TBX15* protein expression in tumor tissues and nontumor tissues.

In our series, the levels of *TBX15* mRNA in tumor tissues were significantly lower compared with those in nontumor tissues (*p* < 0.0001) (Figure 2A). And then, immunohistochemistry revealed that *TBX15* protein expression in tumor tissue was weak compared to that of nontumor tissue (Figure 2B).

### Relationship between *TBX15* mRNA levels and patients’ clinicopathological characteristics

**Figure 3:**
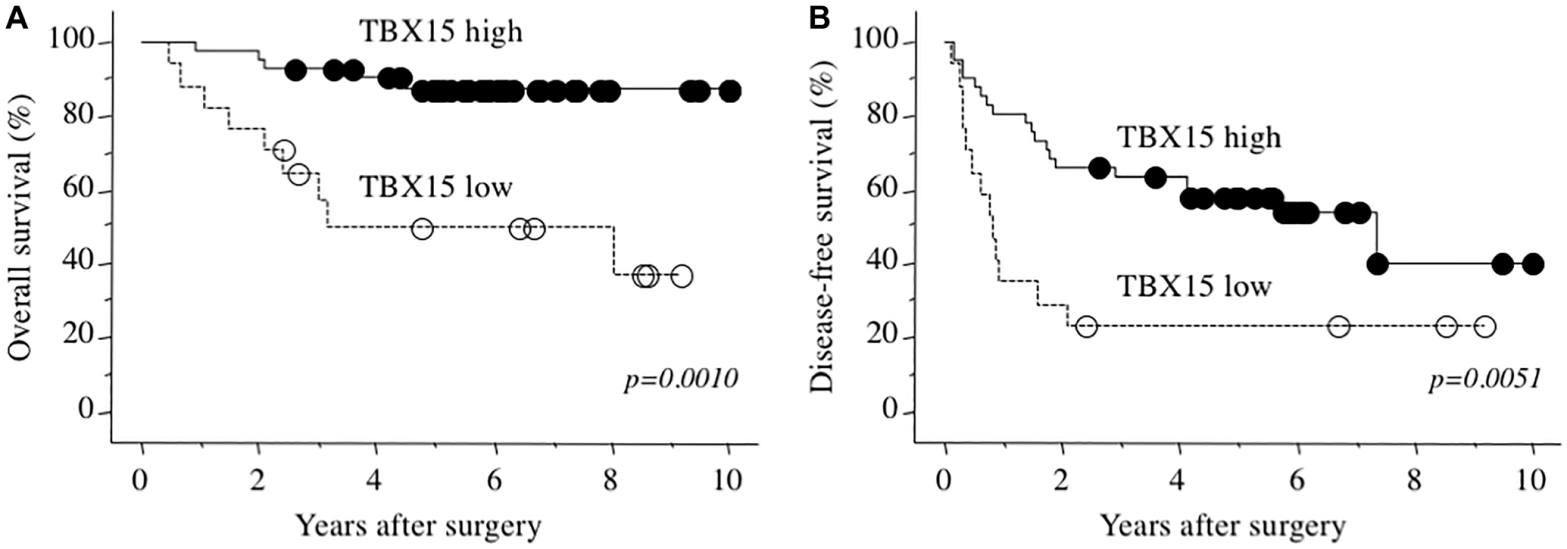
(**A**) Overall survival as a function of *TBX15* mRNA expression. (**B**) Disease-free survival as a function of *TBX15* mRNA expression.

The 3- and 5-years overall survival rates of 58 patients with HCC were 82.6% and 76.8%, respectively. Cutoff value of *TBX15* mRNA for patients’ death was decided by receiver operating characteristics (ROC) curve (AUC 0.727), and Youden’s index was calculated as highest value of 0.4610 at *TBX15* mRNA expression with 0.45457 fold. Therefore, we next allocated the patients according to the levels of *TBX15* mRNA detected in tumor tissues (cutoff value = 0.5-fold) as follows: *TBX15* high-expression group (*n* = 41) and *TBX15* low-expression group (*n* = 17). Table 2 showed the comparison of clinicopathological characteristics according to *TBX15* mRNA expression in tumor tissue. Low *TBX15* expression significantly correlated with higher serum DCP levels, and there were no significant differences between any of the other variables. Notably, there was no significant association regarding the presence or absence of hepatitis virus infection (*p* = 0.8797). The *TBX15* low-expression group experienced significantly worse prognosis compared with that of the *TBX15* high-expression group (*p* = 0.0010), and the 5-year overall survival rates were 43.3% and 86.2%, respectively (Figure 3A). Moreover, the disease-free survival rate of the *TBX15* low-expression group was significantly worse, and the 5-year disease-free survival rates were 23.5% and 58.0%, respectively (Figure 3B).

**Table 2:** Clinicopathological variables and TBX15 mRNA expression in HCC tissues

Factors		TBX15		*p*-value
		Low	High	
		(*n* = 17)	(*n* = 41)	
Age (years)	Median ± SD	66 ± 8.07	68 ± 8.4	0.3836
Gender	Male/Female	16/1	29/12	0.0825
Diabetes mellitus	Absence/Presence	11/6	30/11	0.5403
Hepatic viral infection	Negative/HBV/HCV	5/ 6/6	12/17/12	0.8798
Background liver	NL/CH/LC	3/10/4	7/26/8	0.9339
ICG R15 (%)	≦10 / > 10	12/5	29/12	> 0.9999
Stage	I, II/III, IV	7/10	26/15	0.1511
Maximum tumor size	< 5 cm / ≥ 5 cm	8/9	29/11^*^	0.0780
Number	Single/Multiple	10/7	31/10	0.2211
Growth type	Expandable/Invasive	14/3	35/6	> 0.9999
Differentiation	Well/Others	2/14^*^	6/34^*^	> 0.9999
Portal invasion	Negative/Positive	12/5	32/9	0.7396
Venous invasion	Negative/Positive	14/3	38/3	0.3450
AFP (ng/ml)	≦100 / >100	13/4	26/15	0.3775
DCP (IU/L)	≦300 / > 300	5/12	20/21	0.041

NL: normal liver, CH: chronic hepatitis, LC: liver chirrhosis, ^*^One sample was missing.

### Prognostic factors identified using univariate and multivariate analyses

Univariate analysis identified the number of tumors (multiple) (*p* = 0.0461), growth type (invasive) (*p* = 0.0057), venous invasion (positive) (*p* < 0.0001), serum DCP levels (> 300 IU/L) (*p* < 0.0001), and *TBX15* mRNA levels (Low) (*p* = 0.0010) as significant poor prognostic factors for overall survival (Table 3). When *TBX15* mRNA expression was entered into the proportional hazards model along with number of tumors (multiple), growth type (invasive), venous invasion (positive), and serum DCP levels (> 300 IU/L), low *TBX15* mRNA expression in tumor tissue was identified as one of the independent predictors of poor prognosis (Table 3). Also, in disease-free survival, growth type (invasive) (*p* = 0.0012), venous invasion (positive) (*p* < 0.0001), and low *TBX15* mRNA expression (*p* = 0.0064) were identified as significant independent predictors of poor prognosis (Table 4).

**Table 3:** Prognostic factors of overall survival after curative resection

Factors		5-years survival (%)	*p-*value	Hazard ratio	95% CI	*p*-value
Age (years)	≦70 / >70	81.5/80.1	0.4698			
Gender	Male / Female	74.9/83.1	0.4241			
Diabetes mellitus	Absence/Presence	80.0/69.1	0.5892			
Hepatic viral infection	Negative/HBV/HCV	70.9/76.5/81.9	0.8087			
Stage	I, II/III, IV	87.8/62.6	0.0607			
ICG R15 (%)	≦10 / >10	74.8/81.9	0.9584			
Tumor size (cm)	≦5 / >5	83.4/63.5	0.1775			
Number	Single / Multiple	85.2/57.4	0.0461	1.730	0.452–4.651	0.4657
Growth type	Expandable/Invasive	83.0/41.7	0.0057	11.765	1.984–90.909	0.0078
Differentiation	Well/Others	87.5/78.3	0.9703			
Portal invasion	Negative/Positive	84.0/55.0	0.0507			
Venous invasion	Negative/Positive	84.3/16.7	< 0.0001	1.634	0.309–6.667	0.6451
AFP (ng/ml)	≦100 / > 100	78.7/72.9	0.6964			
DCP (IU/L)	≦300 / > 300	100/59.7	< 0.0001	40.681	2.783–594.731	0.0068
TBX15 mRNA	High/Low	87.2/49.9	0.0006	8.475	2.398–38.462	0.0014

**Table 4:** Prognostic factors of disease-free survival after curative resection

Factors		5-years survival (%)	*p*-value	Hazard ratio	95% CI	*p*-value
Age (years)	≦70 / > 70	48.1/47.7	0.5105			
Gender	Male/Female	46.1/53.8	0.6279			
Diabetes mellitus	Absence/Presence	46.0/52.9	0.7841			
Hepatic viral infection	Negative/HBV/HCV	55.6/47.1/43.5	0.8369			
Stage	I, II/III, IV	57.1/35.2	0.1491			
ICG R15 (%)	≦10 / > 10	48.2/47.1	0.4302			
Tumor size (cm)	≦5 / > 5	50.8/39.4	0.5735			
Number	Single/Multiple	55.9/27.5	0.0868			
Growth type	Expandable/Invasive	52.4/22.2	0.0012	3.106	1.350–6.849	0.0073
Differentiation	Well/Others	50.0/49.3	0.8424			
Portal invasion	Negative/Positive	49.5/42.9	0.6086			
Venous invasion	Negative/Positive	53.3/0	< 0.0001	4.608	1.745–11.364	0.0018
AFP (ng/ml)	≦100 / >100	43.1/57.4	0.2920			
DCP (IU/L)	≦300 / >300	55.5/42.0	0.3295			
TBX15 mRNA	High/Low	58.0/23.5	0.0064	2.079	1.079–4.505	0.0301

## DISCUSSION

The present study presents an array analysis of DNA methylation that identified *TBX15* as the most hypermethylated gene tumor tissue of patients with HCC. These findings indicate that *TBX15* is a candidate regulatory gene that contributes to tumor progression. TCGA data also revealed that *TBX15* hypermethylation was associated with poor prognosis in HCC patients. In our validation set comprising 58 patients with HCC, we found that *TBX15* mRNA expression was significantly reduced in tumor tissues compared with that of nontumor tissues, likely because of *TBX15* hypermethylation. Further, *TBX15* expression did not significantly correlate with tumor status, except for serum DCP levels. In contrast, a significant finding was that relatively low expression of *TBX15* in tumor tissue was an independent prognostic factor for overall and disease-free survival.

The T-box gene family that encodes transcription factors plays diverse, critical roles during embryogenesis [17]. Further, TBX family members contribute to the regulation of the proliferation and differentiation of tissue-specific stem and progenitor cells during organogenesis [17, 18]. Moreover, evidence indicates the association of diverse T-box genes in oncogenesis, tumor invasion, and metastasis of certain cancers [19, 20]. Brachyury, a member of the T-box gene family, induces stemness markers such as NANOG, CD133, CD166, and CD44 in a colorectal cancer cell line by regulating the β-catenin oncogene and contributing to the epithelial-to-mesenchymal transition (EMT) that mediates invasion by tumor cells and metastasis [21]. Regarding the relationships of each subfamily of T-box gene and cancer, *TBX2* and *TBX3* over-expressions were observed in several human cancers, including ovarian, cervical, mammary, liver and pancreatic carcinomas, and current findings revealed its association with malignant behavior [20]. And *TBX2* and *TBX3* over-expressions has been elucidated to induce the tumor growth and metastasis, functioning as downstream target gene of Wnt/beta-catenin and pRb pathway through the suppression of p14^ARF^, p21^CIP1^, NDRG1 and E-cadherin [22, 23]. In contrast, TBX5 induces apoptosis and inhibits the growth of human osteosarcomas and lung carcinomas [24]. Further, *TBX5* may act as a novel tumor suppressor gene, which is inactivated by promoter methylation in colon cancer cells [25]. Detection of *TBX5* methylation may therefore serve as a potential biomarker of prognosis [25].

TBX15 was originally shown to be involved in mesodermal differentiation of the limb skeleton and face as well as in the impairment of adipocyte differentiation [17, 18]. Mutations of *TBX15* are associated with the congenital morphological abnormalities of patients with Cousin syndrome [26, 27]. Recently, the association of *TBX15* and cancer was demonstrated by the detection of breast cancer tissue-specific down-regulation of *TBX15*, suggesting the use of *TBX15* expression as a biomarker to facilitate accurate diagnosis and prognosis or as a predictive marker for treatment efficiency [28]. Further, the *TBX15* promoter is hypermethylated in histological subtypes of ovarian cancer, and the methylation levels and expression of TBX15 inversely correlate [29]. Thus, hypermethylated *TBX15* may serve as a potential biomarker for early detection of the induction and progression of ovarian cancer [29].

In respect to HCC, a study using optimized liquid hybridization capture-based bisulfite sequencing detected tumor-specific hypermethylation of the *TBX15* promoter, suggesting its contribution to hepatocarcinogenesis [30]. Similarly, genome-wide DNA methylation analysis revealed that the promoter region of *TBX15* is hypermethylated and may therefore serve as marker to classify tumor and nontumor tissues [31]. In TCGA data, *TBX15* mRNA expression was significantly lower in tumor tissue of HCC compared to nontumor tissue in HCC patients, as well. However, these reports did not investigate the malignant potential of TBX15.

The activity of TBX15 that may be associated with tumor progression and oncogenesis is unknown, and a few studies have addressed this question. For example, *TBX15* is a methylation marker of prostate cancer associated with advanced stage and ETS-related gene (ERG) expression, which regulates cell proliferation, angiogenesis, and apoptosis [32]. Further, a combination of *TBX15* and other hypermethylated genes is a useful biomarker for ERG expression and recurrence of prostate cancer [30]. TBX15 acts as an anti-apoptotic factor in thyroid cancer by inhibiting the expression of proapoptotic BAX and increasing the expression of antiapoptotic BCL2 and BCL-XL regulators [33]. *TBX15* is up-regulated by TNF-α activation of the NF-κB pathway [34], and the EMT-related gene BMI1 directly represses the expression of *TBX15* in myeloid progenitor cells [35]. Further, TBX15 may interact with corepressors of the Groucho family during mesodermal differentiation [36], which regulate several signaling mechanisms such as the Wingless/Wnt, transforming growth factor-β and NOTCH signaling pathways [37]. These findings suggest an association of TBX15 with oncogenesis in which *TBX15* functions as a tumor suppressor related to tumor progression and resistance to treatment through the EMT, which was regulated by signaling pathways such as Wingless/Wnt [38]. However, TCGA pan cancer analysis of mRNA expression revealed that *TBX15* expressions indicated a various trend according to the types of cancer. Actually, *TBX15* of breast cancer and cholangiocarcinoma were down-regulated compared to normal tissue as well as HCC, meanwhile this gene expressions of other cancer were up-regulated. Hence, this gene might have the pivotal role for tumor malignancy according to the types of cancer, which was possibly related to embryological property.

To further investigate the possible regulatory mechanism of *TBX15* in tumor malignancy of HCC, we performed GSEA according to *TBX15* expression. Notably, the *TBX15* expression-associated signatures “REACTIVE OXYGEN SPECIES (ROS) PATHWAY” was negatively correlated to *TBX1*5 expression with significant displayed considerable normalized enrichment scores (Supplementary Figure 4). To the best of our knowledge, there was no reports in respect with the relationship between *TBX15* and ROS in tumor malignancy. Many reports have demonstrated the pivotal role of ROS in biological processes [39]. Particular in tumor microenviroment, moderate ROS concentration induces cancer cell survival, angiogenesis and metastasis via several cell survival signaling cascade such as MMPs and VEGF, meanwhile high ROS concentration leads to cancer cell apoptosis [39]. In HCC with low *TBX15* expression, ROS production could be moderate and contribute to the worse tumor malignancy. Therefore, ROS targeted therapy might be considered in clinical settings of HCC, because anti-*TBX1*5 therapy was not established in both basic and clinical researches. Further study is still needed to discover the true mechanism for tumor malignancy of *TBX1*5 focused on ROS concentration and its signaling pathway in each cancer. Also, those new findings might be one of clues for the discrepancy which several cancers indicated various *TBX15* expressions.

Together, the present and previous findings support the importance of conducting further investigations to determine the role of *TBX15* in cancer, particularly in tumor progression. However, several limitations must be considered in our study. First, the patient population was very small. Second, selection bias was possible, because of the retrospective one. Third, we did not determine the actual mechanism of *TBX15* for tumor malignancy in basic experiment. Those issues should be solved in several cancers, to clarify the true function of *TBX15* for tumor malignancy in each cancer. In conclusion, reduced expression of TBX15 may serve as a potential biomarker for predicting tumor progression and poor survival as well as a target for antitumor therapy in HCC.

## MATERIALS AND METHODS

### Patients and microarray analysis of DNA methylation

We enrolled 15 patients with NBNC-HCC who underwent hepatectomy at Tokushima University Hospital. The Institutional Review Board of the University of Tokushima Graduate School authorized this study in advance (approved no.: 1800-3), and all patients provided written informed consent.

The DNeasy Blood & Tissue Kit (QIAGEN, USA) was used to extract DNA extraction. Genome-wide DNA methylation status was assessed using an Infinium HumanMethylation450 BeadChip Kit (Illumina, USA) containing 485,764 CpG sites. The DNA methylation profiles of autosomal CpGs in tumor tissue were compared with those of nontumor liver tissues. All analyses were performed according to the procedure provided by the manufacturer. The methylation score assigned to each CpG site is represented by a Beta-value calculated according to the normalized probe fluorescence intensity ratios of methylated to unmethylated CpG sites. Beta-values vary between 0 (fully unmethylated) and 1 (fully methylated). The mean methylation level of each gene (stratified according to upstream region/gene body and CpG island/shore/shelf/nonisland) was calculated as described previously [14].

### Patient selection for gene-expression analysis

For the establishment of our study design, we firstly investigated and compared *TBX15* mRNA expression among NBNC, HBV and HCV patients with HCC who underwent radical resection. Consequently, there were no significant difference *TBX15* mRNA in both tumor and non-tumor tissue among those patients (Supplementary Figure 5). Therefore, in next validation study, we enrolled other 58 patients including NBNC, HBC and HCV patients with HCC without detectable distant organ metastases who underwent radical resection at Tokushima University Hospital, Japan, between April 2004 and March 2011. The 58 patients underwent liver resection as their initial treatment, and curative resection was confirmed according to the findings of pathological examinations. We excluded patients with intrahepatic cholangiocarcinoma such as mixed-type liver cancer. Tumor status, stage and curability were defined according to the Classification of Primary Liver Cancer by the Liver Cancer Study Group of Japan [40]. The 35 men and 13 women ranged in age from 40–81 years (mean, 66.8 years). The age, sex, liver function, hepatitis virus infection, and tumor pathological status were determined.

The patients were followed in the outpatient clinic according to a standard protocol. Briefly, patients were examined every 3 months during the first postoperative year and at least every 4 months thereafter. The levels of α-fetoprotein (AFP) and des-γ-carboxy prothrombin (DCP) were determined during each visit. Dynamic computed tomography of the abdomen was performed every 3 months during the first postoperative year. Recurrence was diagnosed according to computed tomography, magnetic resonance imaging, or both. The mean follow-up of patients was 85.3 months (range, 29.2–140.7 months).

### Immunohistochemistry

Immunohistochemical staining was performed in surgical sections. Tissue specimens were fixed in 10% formaldehyde, embedded in paraffin, and cut into 4-μm-thick sections. We used a rabbit polyclonal anti-TBX15 antibody (1:100, ab115576; Abcam, Cambridge, UK) as a primary antibody. Tissue section was overlaid with 2nd antibody (Dako Real EnVision Kit/HRP, DAKO, Glostrup, Denmark). Peroxidase labeling was developed by incubating in 3,3´ deaminobenzidine tetrahydrochloride. Finally, nuclear counterstaining was completed using Meyer’s hematoxylin solution. Then, sections were dehydrated in alcohol and xylene and enclosed in synthetic resin.

### Quantitative real-time reverse transcription-PCR (qRT-PCR)

The mRNA levels of *TBX15* in tumor and nontumor liver tissues from 58 patients were evaluated using qRT-PCR. Total RNA was extracted using an RNeasy Mini Kit (74106, QIAGEN, Hilden, Germany). Reverse transcription-PCR was performed using a GeneAmp PCR System 9700 and a High Capacity cDNA Reverse Transcription Kit (4368813, Applied Biosystems, Carlsbad, CA, USA). Quantitative RT-PCR was performed using the StepOne Plus Real-Time PCR System and TaqMan Gene Expression Assay (Applied Biosystems). The assay identification number was TBX15 (Hs00537087_ml, Applied Biosystems), and TaqMan human glyceraldehyde-3-phosphate dehydrogenase (*GAPDH*) mRNA served as the control (4326317E, Applied Biosystems). The mRNA expression level was calculated as the ratio of a test sample to that of *GAPDH*.

### TCGA methylation analysis of HCC

We obtained the methylation array datasets of 371 hepatocellular patients from the Broad Institute’s Firehose. The array contained 59 probe sites in TBX15 genomic region. The probe sites were clustered into 3 groups by the methylation beta values, and the methylation score of TBX15 was calculated as the mean of the beta values in each cluster.

### TCGA pan cancer analysis of mRNA expression

We obtained mRNA expression data of 371 hepatocellular patients from the Broad Institute’s Firehose (http://gdac.broadinstitute.org/). We also obtained mRNA expression data of 520 head and neck cancer patients (Head and neck ca.), 501 thyroid carcinoma patients (Thyroid ca.), 1093 breast cancer patients (Breast ca), 176 lung adenocarcinoma patients (Lung adenoca.), 184 esophageal cancer patients (Esophageal ca.), 415 gastric cancer patients (Gastric ca.), 285 colorectal cancer patients (Colorectal ca.), 36 cholangiocarcinoma patients (Cholangio ca.), 178 pancreatic cancer patients (Pancreas ca.), 533 kidney clear cell carcinoma patients (Clear cell renal ca.), 497 Prostate cancer patients (Prostate ca.), 304 cervical cancer patients (cervical), 176 Endometrial carcinoma (Endometrial ca.), 303 ovarian cancer patients (Ovarian ca.) from the Broad Institute’s Firehose. The mRNA expression data was normalized with quantile normalization [41].

### Gene set enrichment analysis (GSEA)

The associations between TBX15 expression and previously defined gene sets were analyzed by gene set enrichment analysis (GSEA) using HCC expression profiles from TCGA dataset. The biologically defined gene sets were obtained from the Molecular Signatures Database v5.2 (http://software.broadinstitute.org/gsea/msigdb/index.jsp).

### Statistical analysis

Statistical analyses were performed using JMP 11.2.0. software (SAS, Campus Drive, Cary, NC, USA). Data are presented as the median ± standard deviation (SD). Statistical significance was defined as *p* < 0.05. Cutoff value of *TBX15* mRNA expression was assessed using Youden’s J statistic with ROC curve. The significance of the relationships between *TBX15* expression and clinicopathological variables were analyzed using the Mann–Whitney and Fisher’s exact tests. Comparisons between more than 3 groups were calculated using one-way ANOVA with the Turkey-Kramer’s test. Survival curves were calculated using the Kaplan–Meier method, and the differences were compared using the log-rank test. To identify independent factors that influenced overall and disease-free survival, variables identified as significant by univariate analysis were included in the multivariate analysis employing the Cox proportional hazards model.

## SUPPLEMENTARY MATERIALS



## Abbreviations

HCC: hepatocellular carcinoma
miRNA: microRNA
LINE-1: long interspersed nuclear element-1
NBNC-HCC: HCC patients without HCV and HBV
TBX15: T-Box 15
NL normal liver; CH: chronic hepatitis
LC: liver cirrhosis
AFP: α-fetoprotein
DCP: des-γ-carboxy prothrombin
ROC: receiver operating characteristics
MMP: matrix metalloproteinases
VEGF: vascular endothelial growth factor
