# Supplementary material for: Reduction of T-Box 15 gene expression in tumor tissue is a prognostic biomarker for patients with hepatocellular carcinoma
Source: Oncotarget. 2020 Dec 29;11(52):4803–12. doi: 10.18632/oncotarget.27852 (PMC7779253; doi:10.18632/oncotarget.27852)
Supplement: Supplementary file 1 [file oncotarget-11-4803-s001.pdf]

Reduction of T-Box 15 gene expression in tumor tissue is a prognostic biomarker for patients with hepatocellular carcinoma

SUPPLEMENTARY MATERIALS

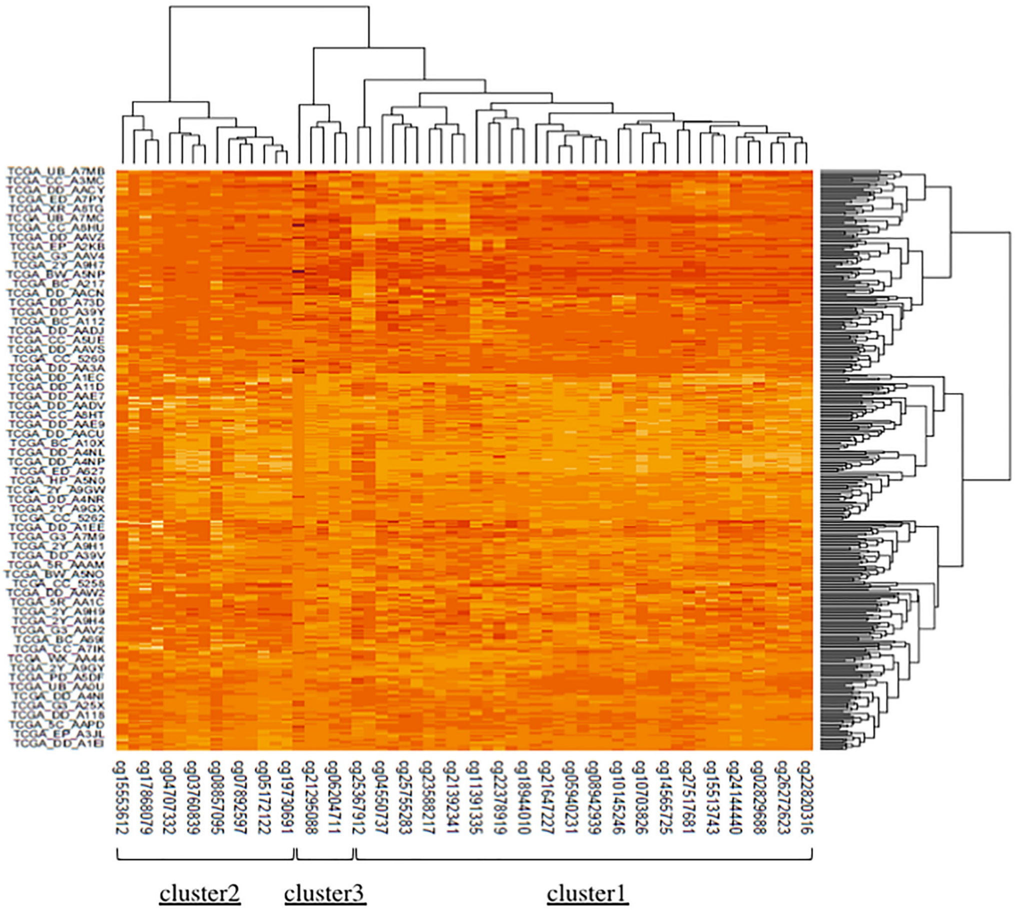

Supplementary Figure 1: Heat map of 59 registered CpG sites of *TBX15* of HCC patients from TCGA dataset.

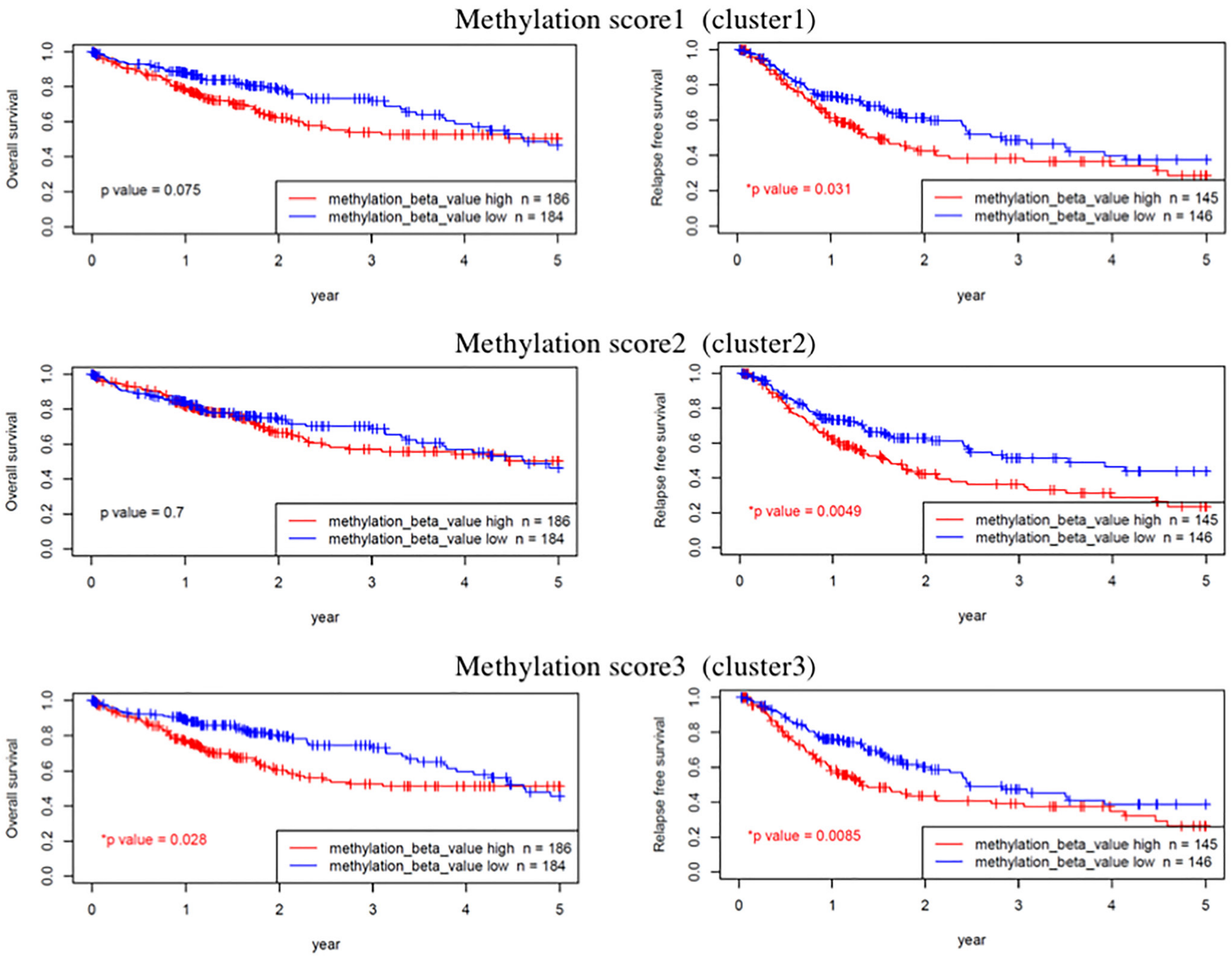

Supplementary Figure 2: Overall and disease-free survival according to 3 clusters classified by beta value.

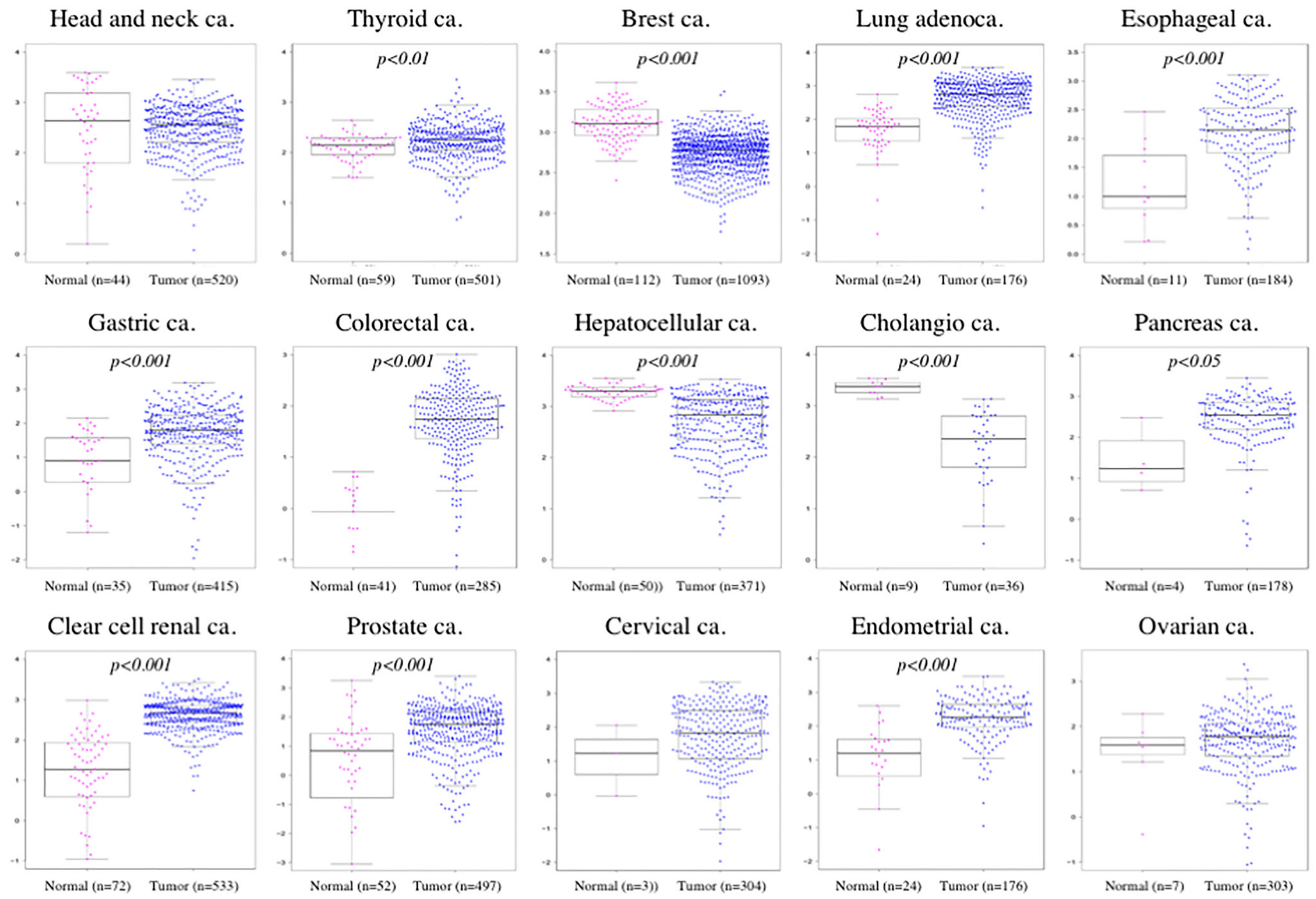

Supplementary Figure 3: *TBX15* mRNA expression of tumor and non-tumor tissue in patients with several types of carcinoma.

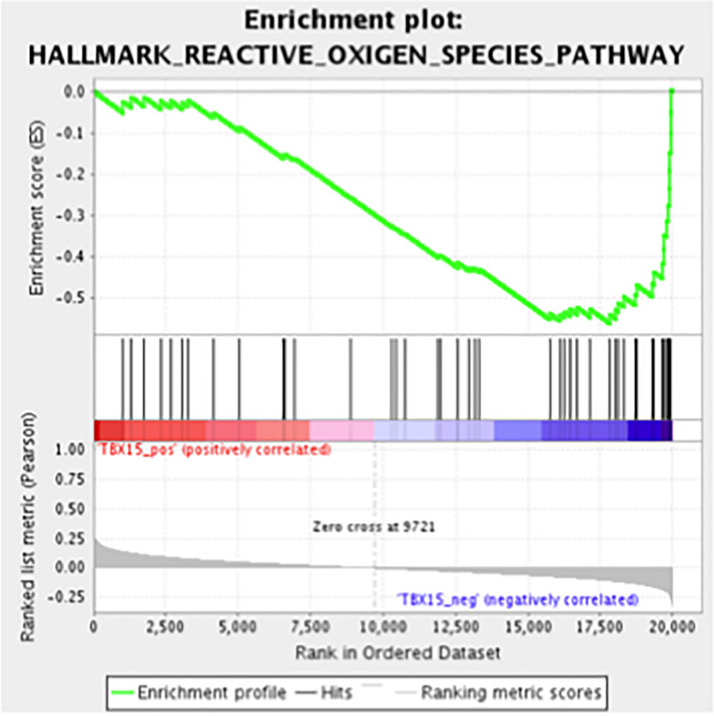

NES = -1.81
FDR = 0.151
p value = 0.016

Supplementary Figure 4: ***TBX15*** **expression-associated signatures using HCC expression profiles from TCGA dataset.**

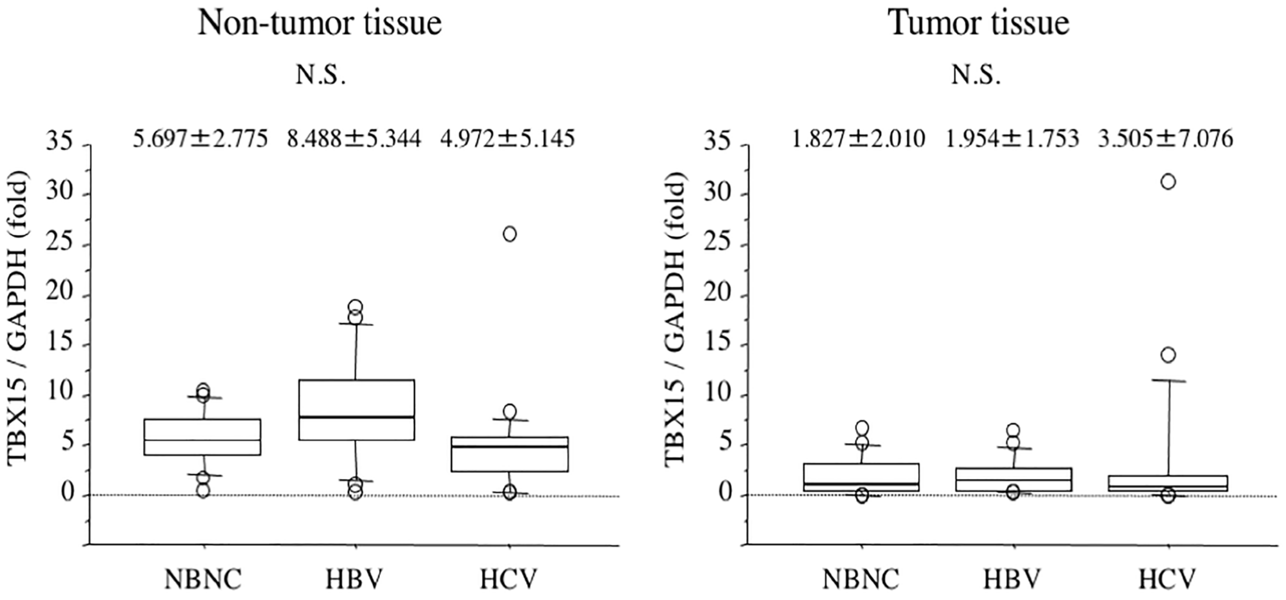

Supplementary Figure 5: *TBX15* mRNA expression of tumor and non-tumor tissue among NBNC, HBV and HCV patients with HCC.
